# Regulation of colonic epithelial cell turnover by IDO contributes to the innate susceptibility of SCID mice to *Trichuris muris* infection

**DOI:** 10.1111/j.1365-3024.2010.01272.x

**Published:** 2011-04

**Authors:** L V BELL, K J ELSE

**Affiliations:** Faculty of Life Sciences, University of ManchesterManchester, UK

**Keywords:** indoleamine 2,3-dioxygenase (IDO), inflammation, large intestine, Trichuris muris

## Abstract

Tryptophan catabolism via the kynurenine pathway is dependent on the enzyme Indoleamine 2,3-dioxygenase (IDO). Expression of IDO is upregulated in a number of inflammatory settings such as wounding and infection, and the resulting local tryptophan depletion may inhibit the replication of intracellular pathogens. *Indo* gene expression is upregulated in the gut during chronic infection with the mouse whipworm *Trichuris muris*. We demonstrate an increase in the rate of colonic epithelial cell turnover after inhibition of IDO in *T.*muris-infected SCID mice, leading to a significant expulsion of parasite burden. We identify the goblet cell as a novel source of IDO and present data revealing a new role for IDO in the regulation of epithelial cell turnover post-infectious challenge.

## Introduction

IDO is a tryptophan-degrading enzyme involved in the initial, rate-limiting step of the kynurenine pathway. IDO is expressed in a number of cell types, such as macrophages, dendritic cells, fibroblasts and endothelial cells (1–3) when inflammation is present; either through normal tissue function or through wounding, infection or tumour growth. It is well documented that IDO induction by IFN-γ, and consequent tryptophan depletion, inhibits the replication of various intracellular pathogens, such as *Toxoplasma gondii* and *Chlamydia pneumoniae* (4–6).

*Trichuris muris* is a naturally occurring gastrointestinal nematode parasite of mice, which is a useful model of *Trichuris trichiura* infection in man. The parasite resides in the caecum and proximal colon, and an IL-4- and IL-13-dependent local Th2 response is required for the host to clear infection (7–9). An important feature of a protective Th2 response is an increase in the rate of epithelial cell turnover, acting to physically force the worm out of its optimal niche within the gut epithelium (10). Some mouse strains, such as BALB/c, mount an appropriate Th2 response and expel worms by day 21 p.i. Conversely, AKR mice mount an inappropriate Th1 response, characterized by increased IFN-γ production, and thus develop a chronic infection (7,8). Immunodeficient SCID mice are also susceptible to *T. muris* and, although lacking in B and T cells, still make IFN-γ in response to *T. muris* infection, with likely cellular sources being the NK cell (11) or macrophage (12).

Epithelial cell turnover is the process by which the intestinal epithelium is constantly renewed. Enterocytes migrate up the crypts whilst proliferating, differentiating and maturing, before being sloughed off into the gut lumen after undergoing programmed cell death (13). Factors that alter the rate of epithelial cell turnover are an under-explored area, but may play important roles in resistance to parasites. For example, neutralization of the chemokine CXCL10 results in an increase in the rate of epithelial cell turnover and a decrease in *T. muris* worm burden in the normally susceptible AKR and SCID mouse strains (10).

It has been shown previously that *Indo* gene expression is upregulated in the gut of both susceptible AKR mice (14) and immunodeficient SCID mice (Datta and Else, unpublished observations). The potential contribution of IDO to the innate susceptibility to *T. muris* infection in SCID mice was investigated using 1-methyl-tryptophan (1-MT). This is a competitive inhibitor of IDO initially used in studies blocking the immune privilege of the placenta (15). Mice treated with 1-MT displayed a marked reduction in worm burden by day 21 p.i., accompanied by a significant increase in the rate of epithelial cell turnover at the site of infection. Immunofluorescence staining indicated concentrated IDO levels in the location of goblet cells within the caecal epithelium. This observation was substantiated using a human goblet cell line, where incubation with IFN-γ or parasite Ag upregulated the expression of *Indo*. We therefore identify the goblet cell as a novel source of IDO. Further, in the absence of adaptive immunity, our data suggest IDO directly regulates the rate of epithelial cell turnover post-infectious challenge.

## Materials and methods

### Mice

SCID mice were bred and maintained by the Biological Services Facility (BSF), University of Manchester, UK in individually ventilated cages. Experiments were conducted in accordance with both the University of Manchester BSF regulations for animal husbandry and with the Home Office Animals (Scientific Procedures) Act (1986).

### Parasite

Maintenance of the *T. muris* life cycle and acquisition of excretory/secretory (E/S) antigen was carried out as previously described (16). Mice were infected with approximately 200 infective eggs by oral gavage, and worm burdens assessed at various timepoints p.i. as previously described (17).

### Immunofluorescence

At autopsy, caecal samples were embedded in optimal cutting temperature medium (R.A Lamb, Eastbourne, UK) and snap-frozen in liquid nitrogen-chilled isopentane (VWR, Leicestershire, UK). Sections with the thickness of 6 μm were cut on a cryomicrotome and IDO^+^ cells identified using the anti-IDO Ab, clone 10·1 (Millipore Ltd, Livingstone, UK). Briefly, this staining protocol involved the use of a mouse on mouse kit containing an Ig blocking reagent and biotinylated anti-Ig secondary Ab (Vector Laboratories Ltd, Peterborough, UK) and a tyramide amplification kit (PerkinElmer, Buckinghamshire, UK), along with an avidin/biotin blocking kit and an ABC detection system. Slides were then mounted in Vectorshield containing Dapi (all Vector Laboratories).

### Histology

Goblet cells were identified using a periodic acid/schiff’s (PAS) staining method on 5 μm sections cut from NBF-fixed, wax-embedded caecal tissue. Briefly, sections were dewaxed in citroclear and rehydrated through decreasing alcohol concentrations, and they were then stained with 1% alcian blue (Sigma-Aldrich, Gillingham, UK) in 3% acetic acid for 5 min, washed and treated with 1% periodic acid (Sigma-Aldrich) for 5 min. After another wash, slides were immersed in Schiff’s reagent (Vickers Laboratories, Pudsey, UK) for 15 min before washing and counterstaining in Harris’ haematoxylin (Raymond A Lamb, Eastbourne, UK) for 1 min. Sections were then allowed to blue in running tap water before being dehydrated and mounted. Slides were then randomized, and goblet cells counted per 20 caecal crypt units (ccu) in three tissue sections per mouse.

### 1-MT

1-MT (Sigma) was dissolved in drinking water at 2 mg/mL to which mice had free access for 1 week preceding, and throughout the course of infection.

### BrdU

Mice were injected with 0·5 mg BrdU (Sigma) in PBS i.p. 40 min or 12 h prior to autopsy. BrdU^+^ cells were then identified by immunohistochemistry in a previously described method (18).

### LS174T cells

LS174T cells were grown in advanced MEM (Invitrogen, Paisley, UK) supplemented with 5% FCS, 2 mm l-glutamine, 100 units/mL penicillin, 100 μg/mL streptomycin (all PAA, Somerset, UK). Cells were plated at 3 × 10^4^ cells/cm^2^ and left overnight before stimulation with *T*. muris E/S (50 μg/mL) recombinant human IFN-γ or IL-13 (both 10 ng/mL; Insight biotechnology, Wembley, UK). Cells were harvested in TRIzol® (Invitrogen) and stored at −80°C until analysed.

### RNA extraction, reverse transcription and quantitative (q) PCR

Mouse IEC or LS174T cells were analysed in a method previously described (19). Expression levels of *Indo* are shown as fold change over levels in naïve mice (for IEC) or unstimulated LS174T cells after normalization to housekeeping gene levels using the ΔΔC^*t*^ method. Primers used for mouse IEC were AGTCCCTGCCTTTGTACACA and GATCCGAGGGCCTCACTAAC for *18S*, CTGCACGACATAGCTACCAGTCTG and ACATTTGAGGGCTCTTCCGACTTG for *Indo*, and primers used for human LS174T cells were CCAACCGCGAGAAGATGAC and GGAAGGAAGGCTGGAAGAGT for *β-actin* and CATGCTGCTCAGTTCCTCCA and CCAGCATCACCTTTTGAAAGGA for *Indo*. All sequences are 5′-3′ starting with the sense primer.

### Statistics

Where two groups were compared, the Mann–Whitney *U*-test was used, and where three or more groups were compared, the Kruskall–Wallis with Dunn’s post-test used, via the statistical package graphpad prism (GraphPad Software, San Diego, CA, USA). A *P* value < 0·05 was considered significant.

## Results

**Figure 1:**
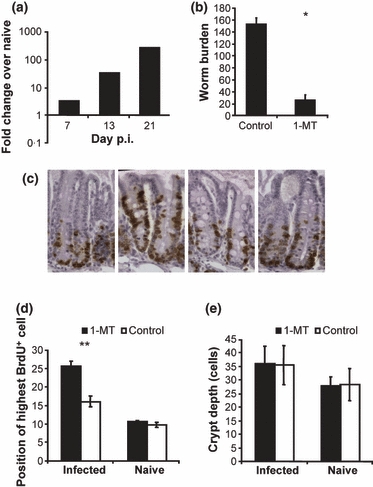
*Trichuris muris*-infected SCID mice express *Indo* in the large intestine, and inhibition of IDO results in a significant parasite expulsion by day 21 p.i., along with an increase in epithelial cell turnover. SCID mice were infected with approx. 200 infective *T. muris* eggs and killed at day 7, 13 and 21 p.i. RNA was extracted from caecal tissue and *Indo* expression analysed by qPCR (a). Data are expressed as fold change over naive mice. Mice were then infected with *T. muris* and treated with 1-MT in drinking water from day −7 throughout infection and killed at day 21 p.i. after having been injected i.p. with 0·5 mg BrdU either 40 min or 12 h earlier. Worm burdens were assessed (b) along with the distance proliferating cells had moved up the crypt axis over a 12-h period (c, d) compared with crypt depth (e) to assess the rate of epithelial cell turnover. Black bars represent 1-MT-treated mice, and white bars represent controls (d and e). All data presented are mean ± SD for five mice per group and are representative of three experimental repeats, *U = 0·00, 5 d.f., *P*=0·0007, **H = 14·64, 4 d.f., *P*=0·0159.

To examine changes in *Indo* expression in the gut in response to *T. muris* infection, SCID mice were infected with *T. muris*, killed at various timepoints p.i. and *Indo* expression analysed from gut samples by qPCR*.* Expression increased p.i over naïve levels up to 522-fold by day 21 p.i. (Figure 1a).

To investigate the functional role of the elevated levels of IDO during *T. muris* infection, SCID mice were treated with the IDO inhibitor 1-MT in drinking water from day −7 to day 21 p.i., at which time mice were killed and worm burdens assessed. Surprisingly, mice treated with 1-MT demonstrated a significant reduction in parasite load by day 21 p.i. compared with control-treated mice (Figure 1b; *U* = 0·00, 5 d.f., *P*=0·0007). To exclude the possibility that 1-MT treatment was directly toxic to the parasite, L1 larvae and adult worms were exposed to 1-MT in an *in vitro* survival assay. Parasites cultured in the presence of 1-MT survived equally as well as those cultured in control conditions over a 12-h period (data not shown). Thus, 1-MT is not toxic to the parasites, and the elevation in IDO levels post-*T. muris* infection of SCID mice contributes to host susceptibility.

One aspect of an effective host Th2 response to *T. muris* is an increase in caecal epithelial cell turnover, which acts to physically drive the worms from their optimal niche at the base of the crypts. A possible role for IDO during *T. muris* infection in susceptible mice may be to downregulate epithelial cell turnover, thereby allowing worms to remain within the caecum of the infected host. To explore this, mice were infected with *T. muris* and treated with 1-MT in drinking water, then injected i.p. with BrdU either 40 min or 12 h prior to killing. BrdU is incorporated into proliferating cells at the base of the caecal crypts, moving up the ‘epithelial escalator’ before eventually shedding into the lumen and can therefore be used to assess the rate of epithelial cell proliferation and turnover. The 40-min timepoint establishes the baseline from which the distance moved up the crypt after 12 h can be calculated. BrdU^+^ cells were detected significantly higher up the crypt axis in *T. muris*-infected mice treated with 1-MT compared with infected, untreated mice (Figure 1c, d; *H* = 14·64, 4 d.f., *P*=0·0159). This increase in epithelial cell proliferation was only observed in *T. muris*-infected mice and not uninfected, 1-MT-treated controls (Figure 1d).

The increase in epithelial cell proliferation was not accompanied by an increase in crypt length (Figure 1e), suggesting that the rate of epithelial cell turnover had increased rather than simply an increase in the proliferation of epithelial cells, which would be associated with a crypt hyperplasia.

**Figure 2:**
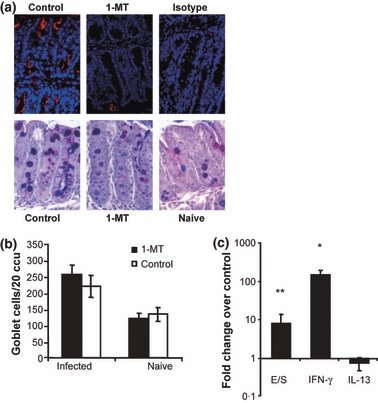
The goblet cell is the major source of IDO in the *Trichuris muris*-infected SCID gut. SCID mice were infected with approximately 200 infective *T. muris* eggs and killed at day 21 p.i. IDO^+^ cells were identified in tissue sections by immunofluorescence (a, red = IDO^+^, blue = DAPI) and goblet cells stained using PAS. All images are at original magnification ×400. Goblet cells were counted per 20 ccu in three tissue sections per mouse, black bars represent 1-MT-treated mice, and white bars represent controls (b). Data are presented as mean ± SD for five mice per group and are representative of three experimental repeats. LS174T cells were stimulated with *T*. *muris* E/S at 50 μg/mL, recombinant human IFN-γ or IL-13 at 10 ng/mL and *Indo* expression assessed by qPCR (c). Data are expressed as fold change over unstimulated cells and are representative of three experimental repeats, *U = 0·00, 5 d.f., *P*= 0·0159 and **U = 0·00, 5 d.f., *P*= 0·0079.

IDO is known to be expressed in macrophages and dendritic cells (20) and has more recently been found to be expressed in epithelial cells (21). Immunofluorescent staining of caecal sections from *T. muris*-infected SCID mice revealed a dense area of IDO^+^ cells in the epithelium, specifically around the location of goblet cells, confirmed by PAS staining (Figure 2a) in infected, control-treated mice. There was very little IDO^+^ staining in the lamina propria, suggesting that the goblet cell, rather than the macrophage or DC may be a source of IDO expression in this model. 1-MT treatment completely abrogated IDO expression in the caecum, but there were no significant differences in goblet cell numbers between 1-MT-treated and untreated infected or naïve mice (Figure 2b).

To confirm the *in vivo* observation that goblet cells are able to express IDO, cells from the human goblet cell line LS174T were stimulated with *T*. *muris* excretory/secretory (E/S) antigen, recombinant human IFN-γ or recombinant human IL-13 and *Indo* expression analysed by qPCR. *Indo* expression was significantly increased in both *T*. muris E/S- and IFN-γ-stimulated LS174T cells over unstimulated cells (*U* = 0·00, 5 d.f., *P*=0·0079 and 0·0159, respectively), whereas IL-13 stimulation resulted in a small downregulation of *Indo* expression (Figure 2c).

## Discussion

In resistant mouse strains, immunity to infection involves the development of a protective Th2 response that mediates worm expulsion at least in part by increasing the rate of epithelial cell turnover, and thus physically forcing the parasite up the epithelial escalator towards the gut lumen (10). IDO is known to inhibit T cell proliferation by local depletion of tryptophan, causing cell-cycle arrest or changes to T cell differentiation and function (2,15,20,22). Inhibitory effects on epithelial cell proliferation and/or the rate of epithelial cell turnover are however not described.

*In vivo* blockade of the chemokine CXCL10 in *T. muris*-infected SCID mice is known to elevate epithelial cell turnover, resulting in worm loss (10). The increase in rate of epithelial cell turnover seen here in SCID mice, in the presence of the IDO inhibitor 1-MT, is therefore likely to underlie parasite expulsion. Thus, the expression of IDO in the gut of *T. muris*-infected SCID mice represents a mechanism that contributes to the regulation of epithelial cell turnover during an infectious challenge. Local induction of IDO by pro-inflammatory cytokines post-infection will deplete local tryptophan and thus suppress epithelial cell proliferation, favouring the survival of *T. muris* within its optimal niche. Therefore, when IDO is inhibited via 1-MT treatment, the resulting increase in available tryptophan may facilitate the increased epithelial cell proliferation and turnover seen, leading to worm expulsion from the large intestine.

IDO expression is induced by IFN-γ in macrophages and DC, as well as fibroblasts and endothelial cells (1–3). IFN-γ levels in the MLN of SCID mice have been shown to increase post-*T. muris* infection (23), and the cellular source of IFN-γ in the SCID mouse is likely to be the NK cell (11) or the macrophage (12). Previous work by Artis *et al.* (23) demonstrated an increase in proliferating cells at the base of the crypt, along with crypt hyperplasia in SCID mice during *T. muris* infection. However, *in vivo* IFN-γ depletion completely abrogated this hyperproliferation, although the rate of epithelial cell turnover was not analysed. Given that IDO is induced by IFN-γ (1–3), the abrogation of hyperproliferation is perhaps surprising, as anti-IFN-γ treatment would be expected to decrease levels of IDO, leading to an increase in epithelial cell proliferation. However, although there is a strong positive correlation between IFN-γ expression and IDO induction, IFN-γ is not essential for the induction of IDO. IDO induction in response to LPS *in vivo* is critically dependent on TNF-α, but not IFN-γ, suggesting that there is an alternative IFN-γ-independent pathway for the induction of IDO (24), and future studies could explore cytokine-mediated regulation of IDO during *T. muris* infection. Of significance in this context is the observation that *T. muris* antigens themselves can directly induce *Indo* expression in a goblet cell line. If these *in vitro* findings are echoed *in vivo*, parasite induction of IDO will facilitate worm survival by suppressing epithelial cell proliferation and turnover.

Goblet cells are a hallmark of many inflammatory responses and have been shown to secrete numerous substances such as mucins, trefoil factors and resistin-like molecule-(RELM)-β (25–27). RELM-β is expressed post-*T. muris* infection and has been shown to disrupt parasite sensory function *in vitro* (28). RELM-β^−/−^ mice infected with a Th1-inducing low dose *T. muris* infection express significantly less IFN-γ and TNF-α in the gut than control mice, fail to induce intestinal inflammation and do not sustain infection (29). The decrease in both IFN-γ and TNF-α in the RELM-β^−/−^ mice would limit IDO induction, and their resistant phenotype may thus in part be associated with an elevation in local tryptophan levels and increased epithelial cell turnover.

IDO has been shown to play a role in many biological settings, such as during pregnancy, inflammation, microbial infection and cancer and may therefore become a potential therapeutic target for the treatment of a wide range of chronic diseases. We report here for the first time an important role for IDO in the control of innate susceptibility of SCID mice to intestinal nematode infection. We show that IDO is able to regulate gut homoeostasis during an infectious challenge by dampening down the rate of epithelial cell turnover and identify the goblet cell as a novel source. As has been shown for protozoan parasites, induction of IDO by pro-inflammatory cytokines may reflect a response by the host to eliminate infection by starving the parasite of the essential amino acid tryptophan. However, in the context of intestinal nematode infection, IDO induction is actually detrimental to the host, as tryptophan is required to increase the epithelial cell turnover, which in turn leads to parasite expulsion. The induction of IDO by parasite-derived antigens may therefore represent a novel parasite survival strategy. Thus, identifying the component(s) of the *T. muris* E/S antigens that promote IDO expression becomes a significant priority, and our data suggest that the development of vaccines that target the IDO-inducing parasite antigens will facilitate worm expulsion.

## Abbreviations:

1-MT: 1-methyl-tryptophan
ccu: caecal crypt units
E/S: excretory/secretory
IDO: Indoleamine 2,3-dioxygenase
IEC: intestinal epithelial cells
